# *Plasmodium vivax* Gametocytes Adherence to Bone Marrow Endothelial Cells

**DOI:** 10.3389/fcimb.2021.614985

**Published:** 2021-06-24

**Authors:** Luis Carlos Salazar Alvarez, Omaira Vera Lizcano, Dayanne Kamylla Alves da Silva Barros, Djane Clarys Baia-da-Silva, Wuelton Marcelo Monteiro, Paulo Filemon Paolluci Pimenta, Marcus Vinicius Guimarães de Lacerda, Fabio Trindade Maranhão Costa, Stefanie Costa Pinto Lopes

**Affiliations:** ^1^ Fundação de Medicina Tropical Dr. Heitor Vieira Dourado (FMT-HVD), Manaus, Brazil; ^2^ Programa de Pós-Graduação em Medicina Tropical, Universidade do Estado do Amazonas, Manaus, Brazil; ^3^ Laboratório de Doenças Tropicais Prof. Dr. Luiz Jacintho da Silva, Departamento de Genética, Evolução, Microbiologia e Imunologia, Universidade de Campinas - UNICAMP, Campinas, Brazil; ^4^ Grupo de Investigación QUIBIO, Facultad de Ciencias Básicas, Universidad Santiago de Cali, Cali, Colombia; ^5^ Instituto Leonidas & Maria Deane - ILMD/Fiocruz Amazônia, Manaus, Brazil; ^6^ Instituto René Rachou - IRR/Fiocruz Minas, Belo Horizonte, Brazil

**Keywords:** Malaria, *Plasmodium vivax*, cytoadhesion, bone marrow, gametocytes

## Abstract

In a *Plasmodium vivax* infection, it was shown a proportionally increased on gametocyte distribution within the bone marrow aspirant, suggesting a role of this organ as a reservoir for this parasite stage. Here, we evaluated the *ex vivo* cytoadhesive capacity of *P. vivax* gametocytes to bone marrow endothelial cells (HBMEC) and investigated the involvement of some receptors in the cytoadhesion process by using transfected CHO cells (CHO-ICAM1, CHO-CD36 and CHO-VCAM), wild type (CHO-K1) or deficient in heparan and chondroitin sulfate (CHO-745). *Ex-vivo* cytoadhesion assays were performed using a total of 44 *P. vivax* isolates enriched in gametocyte stages by Percoll gradient in the different cell lines. The majority of isolates (88.9%) were able to adhere to HBMEC monolayer. ICAM1 seemed to be the sole receptor significantly involved. CD-36 was the receptor with higher adhesion rate, despite no significance was noticed when compared to CHO-745. We demonstrated that gametocyte *P. vivax* adheres *ex vivo* to bone marrow endothelial cells. Moreover, *P. vivax* gametocytes display the ability to adhere to all CHO cells investigated, especially to CHO-ICAM1. These findings bring insights to the comprehension of the role of the bone marrow as a *P. vivax* reservoir and the potential impact on parasite transmission to the vector.

## Introduction

Malaria continues to be an important public health problem despite global efforts to control it. *Plasmodium vivax* malaria is the most widely distributed type of malaria in the world, positioning at risk of infection 2.5 billion of individuals (Gething et al., 2012). It occurs mostly in Latin America, South and Southeast Asia, Korea, Southwest China, the Middle East, and some endemic regions in Africa (Guerra et al., 2010). Several efforts have been done to control malaria in the globe. However, *P. vivax* uniqueness features in terms of its biology have hampered this strategy, especially the rapid appearance of gametocytes into the peripheral blood (McKenzie et al., 2007). Gametocytes have been detected in bone marrow (BM) aspirates from *P. vivax* isolates collected in different endemic areas (Baro et al., 2017). Recently, in 14 BM autopsies from non-human primates infected with *P. vivax*, an expressive amount of gametocytes was detected accumulated/sequestered, suggesting the role of this organ as a parasite reservoir (Obaldia et al., 2018).

Indeed, in *Plasmodium falciparum* infections, immature gametocytes have the ability to cytoadhere to different models of endothelial cells by the interaction with endothelial receptors CD36 and ICAM1 (Rogers et al., 1996). A post-mortem study of *P. falciparum* infections identified a greater number of gametocytes in the BM in comparison with other organs, suggesting the sequestration of gametocytes in this organ and postulating it a niche for gametocytogenesis in *P. falciparum* infections (Joice et al., 2014). The results of these studies provide theoretical bases in the understanding of gametocytogenesis, which could allow the development of new interventions to block parasite transmission to the vectors in *P. falciparum* infections (Rogers et al., 2000; Tiburcio et al., 2013; Aguilar et al., 2014; Joice et al., 2014).

*P. vivax* invades reticulocytes that are prevalent in the bone marrow parenchyma (Malleret et al., 2015), and gametocytes and young parasite stages have been detected in proportionally increased distributions within the marrow aspirates of a clinical case (Mayor and Alano, 2015). These observations indicate a role of the bone marrow in *P. vivax* infection. Recent studies on *P. vivax* infections have exposed BM as a potential niche for the development of this species (Malleret et al., 2015; Mayor and Alano, 2015; Baro et al., 2017), exhibiting similar behavior to the *P. falciparum* infections. Considering the difficulty in performing BM biopsies to see *in vivo* adherence of iRBCs, in the present study, we evaluated the cytoadhesive capacity of *P. vivax* gametocytes *in vitro* in BM endothelial cells (HBMEC). In parallel, we evaluated the potential receptors involved in the adhesion of gametocytes through the use of CHO-K1 endothelial cells transfected with the ICAM1, VCAM and CD36 receptors. CHO-745 cell line was used like control; this cell line does not express any of these receptors.

## Materials and Methods

### Sample Collection

Samples were collected at the *Fundação de Medicina Tropical Dr. Heitor Vieira Dourado* (FMT-HVD), located in the city of Manaus, Amazonas state, Brazil. Forty-four patients with *P. vivax* positive thick blood smears with parasitemia greater than or equal to two crosses (11-100 parasites per 100 thick-film field) and with no current antimalarial treatment were included in the study. Before treatment, a sample of 10 mL peripheral blood was collected with the heparin coated Vacutainer tubes. Patients were treated following the Brazilian Health Ministry guidelines (chloroquine plus primaquine 0.5mg/kg/day for 7 days) (Ministério da Saúde do Brasil, 2010).

### *P. vivax* Gametocyte Purification

Blood was immediately processed to obtain enriched *P. vivax* gametocytes- parasitized red blood cells (gPv-pRBC). Blood was centrifuged and red blood cells (RBC) cells were washed twice with RPMI-1640 medium (SIGMA-ALDRICH) and passed through a cellulose (SIGMA-ALDRICH) column to remove leukocytes and platelets as previously described (Russell et al., 2011). A Percoll (GE HEALTHCARE) cushion (45%) was performed to separate the mature asexual parasites and gametocytes from the younger asexual forms and non-infected erythrocytes as described elsewhere (Vera et al., 2015). Parasitemia and gametocytemia (the percentage of parasites in the gametocytes stage) were determined in thin blood smears stained with Giemsa after the purification process. For this purpose, 200 parasites were counted and the parasite stage determined by an experienced microscopist. Representative photos of parasite stage determination were presented in **Supplementary Figure 2**.

### Endothelial and CHO Cells Culture

Human bone marrow endothelial cell (BMEC-1/CDC) were kindly provided by Jason Goldstein from Center for Disease Control and Prevention. BM endothelial cells (passage 18 to 25) were grown with MCDB 131 medium GIBCO (THERMO FISHER SCIENTIFC) supplemented with 1 ug/mL hydrocortisone, 10ng/mL epidermal growth factor and 10% fetal bovine serum (Candal et al., 1996).

Chinese Hamster Ovary Cell (CHO) K1 and stable transfectants of CHO expressing CD36, ICAM1 and VCAM and the CSA negative CHO variant *pgsA* (CHO-745) (Gölnitz et al., 2008) were cultured in RPMI 1640 containing 10% fetal bovine serum in a 5% CO_2_ atmosphere at 37°C. These cells are kindly provided by Dr. Gerhard Wunderlich from Universidade de São Paulo (USP).

### *P. vivax* Adhesion Assay

HBMEC, CHO-K1, CHO-745, CHO-CD36, CHO-ICAM1 and CHO-VCAM cells were grown in four-well slides (Culture Slides, BD BIOSCIENCES) until reaching 80-90% of confluence. Then, 5x10^4^ gPv-pRBC were added to the cell monolayer in a total volume of 200µL in adhesion medium (RPMI 1640 pH 6.8, SIGMA-ALDRICH) and the slides were incubated for one hour at 37°C. After the incubation, non-adhered parasites were removed by gravidity by washing three times the slides with adhesion medium. Subsequently, cells were fixed with methanol and stained with Giemsa.

For the cytoadhesion assays in HBMEC, two wells of the four were stimulated with TNF-α (10 ng/mL) for 18 h previously to parasite addition in order to simulate an inflammatory environment and induce the expression of cell adhesion molecules.

Rates of gPv-pRBC adherence to different cell types were determined by light microscopy and the results were expressed as the mean ± standard deviation of the number of adhered gametocytes per well. Each isolate was checked for the adhesion ability in duplicate (two wells) for the evaluated cell.

### Flow Cytometry

ICAM1 and CD36 expression were analyzed by FACS. Briefly, cells were detached by scraping and 5x10^5^ cells were used to perform the staining. First the cells were washed twice with 1X PBS supplemented with 5% fetal bovine serum (Blocking Solution) and then cells were blocked for 30 min at 4°C. After incubation, cells were spun at 1500 RPM x 5 min and then the cells were incubated with the antibody for 1h at 4°C following the manufacturer’s instructions (FITC mouse anti-human CD36, clone CB38 and PE mouse anti-human CD54, clone LB-2 PE; BD BIOSCIENCES). The cells were washed three times with 1X PBS and resuspended in a volume of 500 µL. The mean fluorescence intensity was evaluated using a BD FACSVerse (BD BIOSCIENCES).

### Statistical Analysis

The data were examined using the Shapiro-Wilk normality test. The Kruskal-Wallis and Wilcoxon signed-rank tests were used when required. The differences were considered statistically significant when *P*<0.05. The statistical analyses were performed using the GraphPad Prism software, version 7.0. (GRAPHPAD SOFTWARE, CA).

## Results

A total of 44 samples was used for the evaluation of *P. vivax* gametocytes adhesion capacity, 18 isolates were used in adhesion assay in HBMEC cells and the other 26 isolates used in the adhesion assay of the different receptors in CHO cells (**Supplementary Figure 1**). After purification, parasitemia and gametocytaemia obtained were 69.9% (ranging from 49% and 89.9%) and 26.4% (ranging from 4% and 80%), respectively. The majority of isolates showed gPv-pRBC cytoadhesive capacity with 94.4% (17/18) of the tested isolates being able to cytoadhere in static condition to HBMEC as showed in the representative photomicrograph (**Supplementary Figures 2A–C**). Furthermore, it was observed that the asexual phases (trophozoite and schizonts) have also been able to adhere to HBMEC cells (**Supplementary Figure 2D**) and have presented a greater adhesion capacity compared to gametocytes in the HBMEC cells (*P* = 0.0156) (**Supplementary Figure 3**).

**Figure 1:**
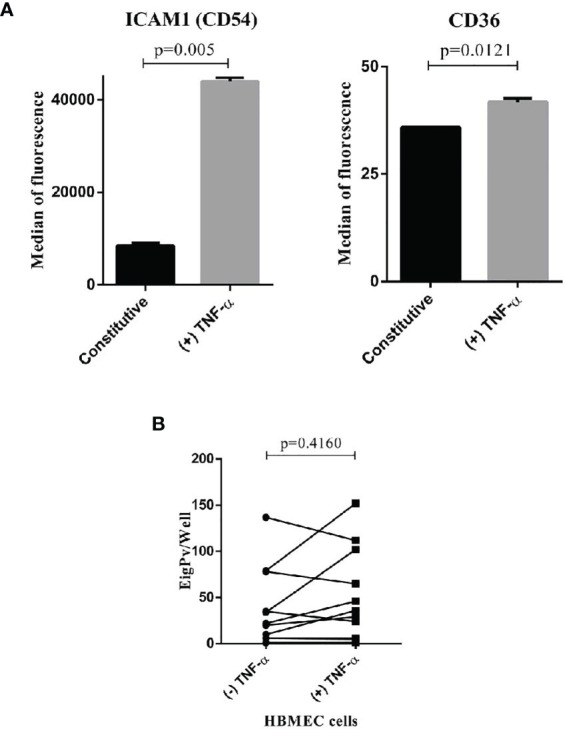
*Plasmodium vivax* gametocytes adhesion to HBMEC. **(A)** Expression of ICAM1 and CD36 in HBMEC cell with or without stimulation of TNF-alpha. The cells were incubated with TNF-alpha (10 ng/mL) for 18h stimulated the expression of endothelial cells markers ICAM1 and CD36. The expression was determinate for FACS and expressed as the median of fluorescence. The data were analyzed by Unpaired t test. **(B)** Cytoadhesion assays of *P. vivax* gametocytes to bone marrow endothelial cells (HBMEC) with or without stimulation of TNF-α. The cytoadhesion was expressed as the total number of gPv-pRBC adhered to HBMEC cells per slide well. The data are shown as the mean ± standard deviation from a total of 13 biological replicates (isolates). The data were analyzed by Wilcoxon Rank test.

Of the 18 isolates evaluated in the HBMEC cells, only 13 isolates were evaluated simultaneously in the two conditions (with or without TNF- α), (**Supplementary Figure 1**). The remaining five isolates were evaluated in a single condition as shown in **Supplementary Table 1**. The analysis of the 13 isolates showed cytoadhesion rate found in this cell was 71.6 ± 135.8 gPv-pRBC adhered per well (ranging from 0 to 679). TNF-α stimulation did not increase the gPv-pRBC cytoadhesion rate in HBMEC cells (*P* = 0.4160; Wilcoxon signed-rank test) (**Figure 1B**). Even though the TNF-α stimulation has increased the expression of ICAM1 and CD36 receptors on HBMEC surface (**Figure 1A**).

**Figure 2:**
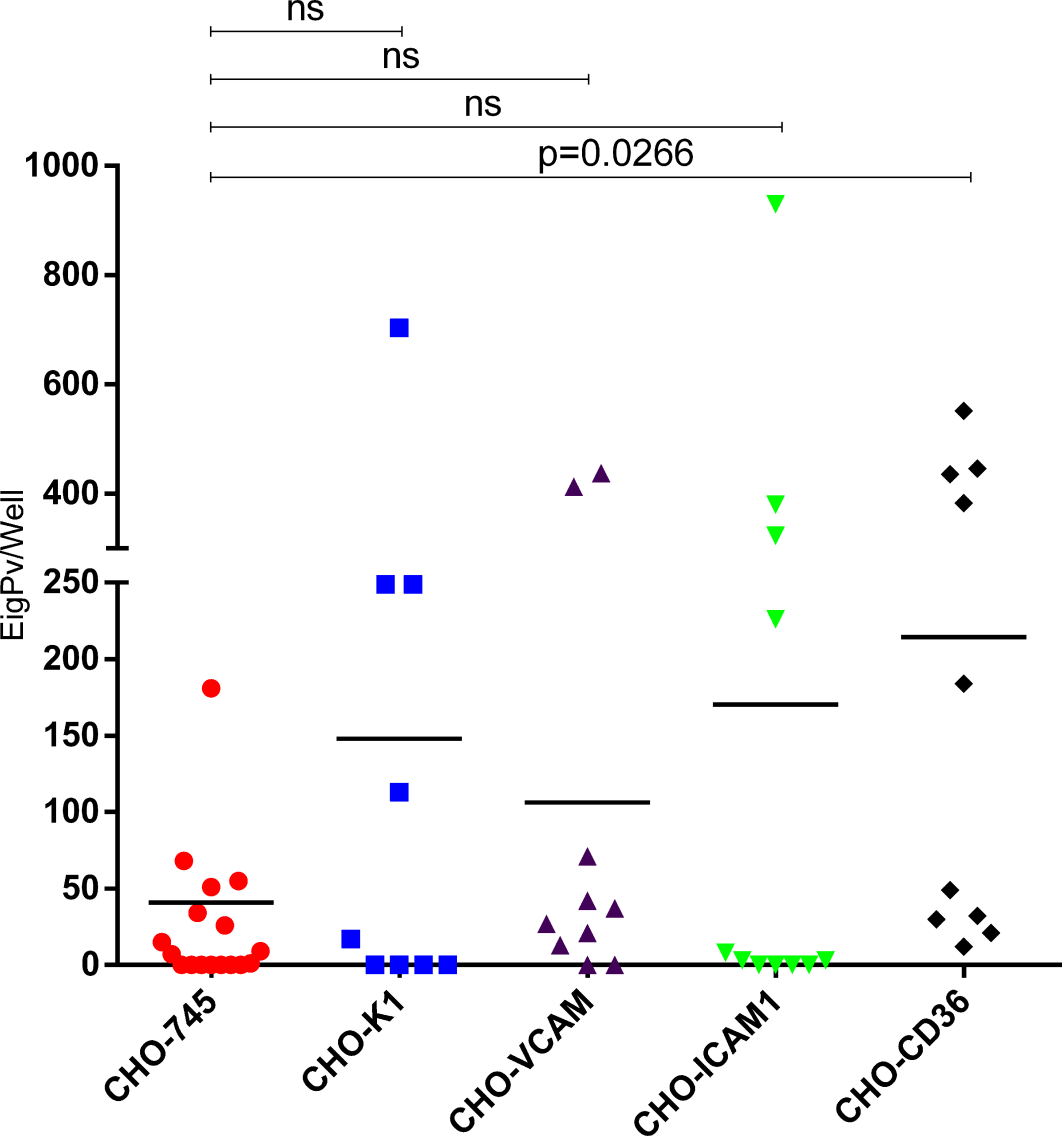
*Plasmodium vivax* gametocytes adhesion to different endothelial receptors. Cytoadhesion assays in CHO cells K1 and CHO-745 (CHO-K1 depleted of glycosamynoglycans) and CHO-745 transfected with the endothelial receptors VCAM, ICAM1 and CD36. Cytoadhesion was expressed as total gPv-pRBC adhered to CHO cells per well. Each symbol represents the mean adhesion rate of one isolate to the analyzed cell. The black line is the mean adhesion rate of all isolates evaluated to the respective CHO cell. The data were analyzed by Kruskall Wallis and Dunns post test. ns, not significant.

Assays in CHO cells demonstrated that gPv-pRBC have the ability to cytoadhere to all CHO cells evaluated. However, the percentage of isolates that adhere to each receptor varied as shown in **Supplementary Table 2**. All tested isolates have shown gPv-pRBC adhesion in CHO-CD36 cells (10/10), whereas only 61.1% of isolates have being able to adhere to CHO-745 (11/18) and 55.5% to CHO-K1 (5/9). In CHO-ICAM1 cells, 63.6% (7/11) of the isolates tested have presented gPv-pRBC adhesive capacity, while 80% (8/10) of the isolates have gPv-pRBC adhered to CHO-VCAM cells. The higher cytoadhesion rate was found for CHO-CD36 (214.5 ± 215.8), followed by CHO-ICAM1 (170.3 ± 290.85), CHO-K1 (148.0 ± 233.27), CHO-VCAM (106.2 ± 169.68) and lastly, CHO-745 (40.8 ± 75.98) as expected. We found significantly difference only in the mean gPv-pRBC adhesion rate to CHO-CD36 (*P* = 0.0266; Kruskall Wallis and Dunn’s post test) in comparison with the mock cell (CHO-745) (**Figure 2**).

**Figure 3:**
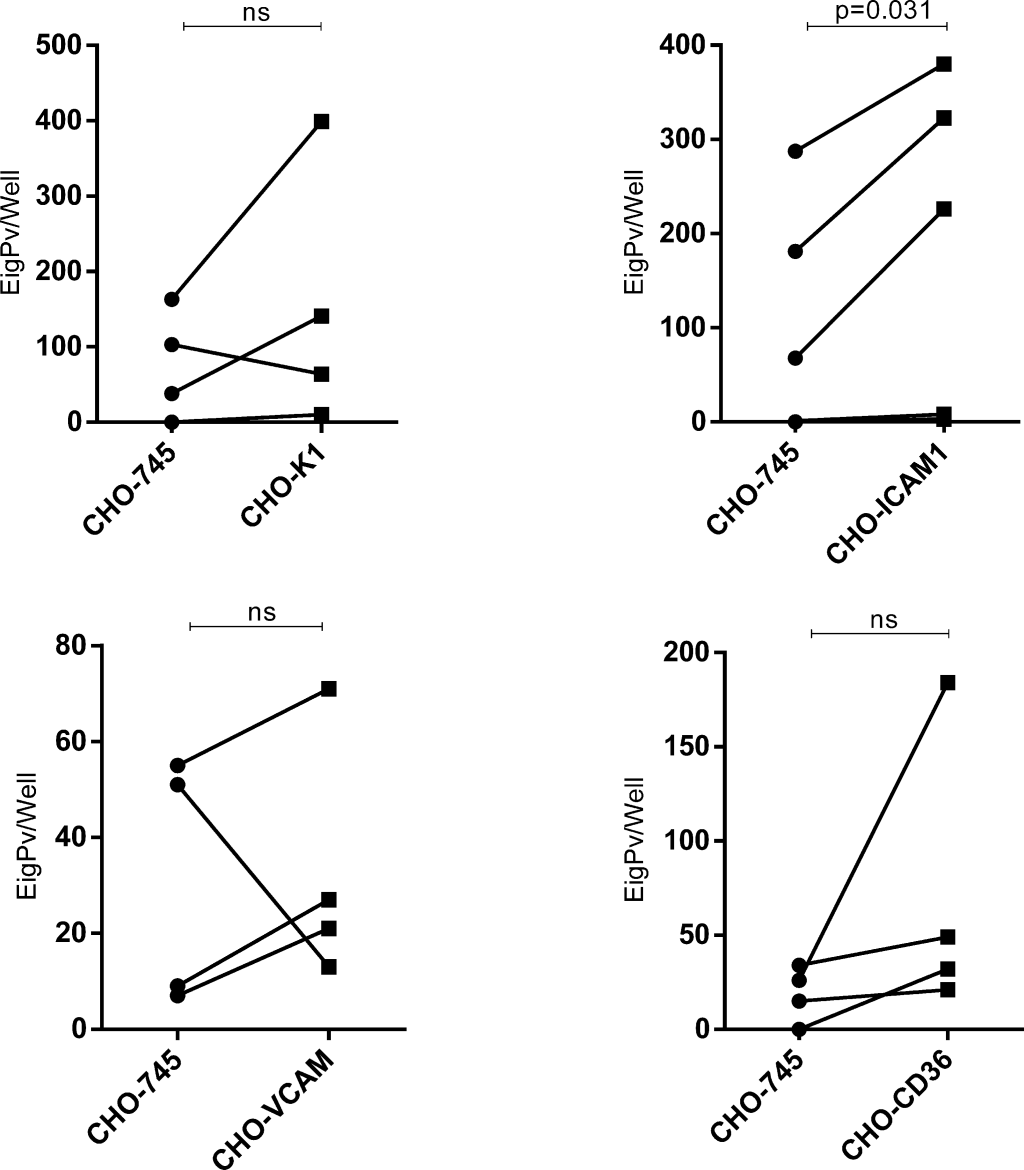
Comparison between adhesive capacities of *Plasmodium vivax* gametocytes to different endothelial receptors. Adhesion ability to different endothelial receptors of *Plasmodium vivax* gametocytes from one isolate was compared to its ability to adhere to the control cell (CHO-745). Cytoadhesion was expressed as total gPv-pRBC adhered to CHO cells per well. The gametocytes adhered per well from each isolate are presented as a circle (adhesion to CHO-745) or square (adhesion to transfected CHO) and linked by a line. The data was analyzed for Wilcoxon signed-rank test (P<0.05). ns, not significant.

Afterwards, we performed a paired comparison of the cytoadhesion ability of the same gPv-pRBC isolate in CHO-745 cells versus specific endothelial receptor (CD36, ICAM1, VCAM and glycosaminoglycans in CHO-K1) in 4 isolates each. It was found a difference in gPv-pRBC cytoadhesion ability only in ICAM1 transfected CHO cells (*P* = 0.031; Wilcoxon signed-rank test) (**Figure 3**).

## Discussion

Our results demonstrated that the cytoadhesive capacity of *P. viv*ax is not restricted to the asexual phases, but for the first time, it is shown that *P. vivax* gametocytes have the ability to *ex vivo* cytoadhere to BM endothelial cells. Recently, Baro et al. (2017) described a morphological and molecular study of BM aspirates from a *P. vivax* patient with an unusually high parasitemia in Manaus, Amazonas, Brazil. On admission, ring- and schizont-infected cells and gametocytes were significantly more abundant in the BM. Obaldia et al. (2018) present a study of *P. vivax* stage distributions in infected tissues of non-human primate (NHP) malaria models as well as in blood from human infections and histological data demonstrated a major fraction of gametocytes in the parenchyma of BM, whereas asexual schizont forms are enriched to a lesser extent in this region of the bone marrow as well as sinusoids of the liver. Smaller fractions of gametocytes and schizonts are represented in the blood circulation. These findings corroborate the hypothesis that subpopulations of asexual and gametocyte stages accumulate/sequester otherwise than the circulating blood *during P. vivax* infection, similar to previous observations in *P. falciparum* (Joice et al., 2014).

We herein demonstrated that gPv-pRBC exhibits the adhesive capacity to HBMEC cells independently of the presence of the TNF-α stimulus. Therefore, the endothelial receptors involved in gPv-pRBC adhesion are probably constitutively expressed in this cell. Although an increase in ICAM1 receptor expression was identified after to stimuli whit TNF-α, we did not observe an increase in the cytoadhesion capacity of *P. vivax* gametocytes, thus presenting a different behavior than the adhesion capacity of gametocytes in *P. falciparum* infections, where a greater adhesion capacity is identified in cells stimulated with TNF compared to cells without stimulation (Rogers et al., 2000; Silvestrini et al., 2012).

In relation to the *P. vivax* cytoadhesion receptors, studies have revealed the participation of the ICAM1 receptor and chondroitin sulfate A (CSA) (Carvalho et al., 2010; De las Salas et al., 2013). Our results show that ICAM1 probably plays an important role in the cytoadhesion process of *P. vivax* gametocytes and it has similar behavior in the cytoadhesion phenomenon of the asexual phases exposed in previous works (Carvalho et al., 2010). Importantly, young reticulocytes (CD71high) were also demonstrated to adhere to CHO-ICAM1 transfected cells (Malleret et al., 2013). Assays in CHO cells transfected or not, demonstrated that *P. vivax* gametocytes have a variable cytoadhesive capacity. The variability may be due to the genetic diversity of the human samples, which differs from studies in *P. falciparum* infections that uses *in vitro* parasite cultures, which allows greater control of the genetic variability of the parasite evaluated. However, some isolates showed a high adhesion rate even in CHO depleted cells of glycosaminoglycans (CHO745) revealing that other receptors besides those evaluated here may be involved in this process.

When the gPv-pRBC adhesive capacity was evaluated independently of the individuality of each isolate, we found that there was a greater adhesion of gPv-pRBC to the CD36 receptor compared to the other receptors tested. This behavior was observed in other studies in which *P. falciparum* gametocytes (I and IIA) presented the greater adhesive capacity to this receptor (Crandall et al., 1994; Rogers et al., 1996). In *P. vivax*, a previous study did not observe the involvement of CD36 in the adhesion ability of the asexual form (Carvalho et al., 2010). Although, when we performed a paired comparison (comparing the adhesion ability of the same isolate) we found only a significant difference in the adhesion to CHO-ICAM1 in relation to the mock cell CHO-745. However, we could not rule out the involvements of the other receptors studied, since a limited number of isolates were evaluated for each receptor.

On the other hand, we must highlight that our observations have been generated from purified peripheral blood gametocytes, suggesting the presence of a larger population of gametocytes in the mature stage. Different studies have shown that the adhesion capacity of gametocytes in *P. falciparum* infections is correlated to their level of maturity. Immature gametocytes have the ability to adhere and then they are sequestered mostly in the bone marrow. Modifications in membrane proteins and morphological changes during maturation culminates in the loss of adhesive capacity of the mature gametocytes and egress from the bone marrow to peripheral blood. Then *P. falciparum* gametocyte adhesive properties are involved in the dynamics of the gametocytogenesis process (Silvestrini et al., 2012; Tibúrcio et al., 2012; Tiburcio et al., 2013; Messina et al., 2018). Furthermore, a recent analysis of the distribution of the different phases of *P. vivax* in a non-human primate (NHP) model, revealed a higher concentration of immature gametocytes in the bone marrow, corroborating that circulating gametocytes are potentially in more mature stage and this could influence its adhesion capacity to host cells (Obaldia et al., 2018). However, to date there are no studies that allow us to correctly categorize the maturity phase for *P. vivax* gametocytes

Here we demonstrated that gPv-pRBCs are able to adhere to BM cells. This organ is believed to be a place where gametocytogenesis occurs in some *Plasmodium* species. In addition, gPv-pRBCs showed the ability to adhere to all CHO cells evaluated, with the greater adhesive capacity to CHO-ICAM1 cells compared to CHO-745 control cells, pointing out the involvement of this receptor in the adhesion process. However, our data did not exclude that other receptors could be also involved in this phenomenon. Therefore, more studies should be carried out to comprehend the role of this phenomenon in parasite survival and transmission to the vector.

## Data Availability Statement

The original contributions presented in the study are included in the article/**Supplementary Material**. Further inquiries can be directed to the corresponding authors.

## Ethics Statement

The studies involving human participants were reviewed and approved by Ethical Board of Fundação de Medicina Tropical Dr. Heitor Vieira Dourado (CAAE: 54532416.0.0000.0005, approval number 1.591.958). The patients/participants provided their written informed consent to participate in this study.

## Author Contributions

LS, DAS, OV, and SL were responsible for *P. vivax* adhesion assay and sample organization. LS, SL, and OV performed gametocytes purification. LS, WM, and SL helped with statistical analysis. LS, DBS, ML, PP, FC, WM, and SL helped draft the manuscript and manuscript revision. LS, ML, FC, PP, WM, and SL participated in study design, coordination, and writing the final version of the manuscript. All authors contributed to the article and approved the submitted version.

## Funding

Fundação de Amparo á Pesquisa do Estado de São Paulo (FAPESP) (Grant 2017/18611-7), Conselho Nacional de Desenvolvimento Científico e Tecnológico (CNPq) (Grant 301795/2013-4) and Fundação de Amparo á Pesquisa do Estado do Amazonas (FAPEAM) (PRÓ-ESTADO). LS, DAS, OV and DBS received scholarships from one of the following Brazilian agencies: Coordenação de Aperfeiçoamento de Pessoal de Nível Superior (CAPES), FAPEAM, FAPESP and CNPq. 

## Conflict of Interest

The authors declare that the research was conducted in the absence of any commercial or financial relationships that could be construed as a potential conflict of interest.

## Abbreviations

BM, Bone Marrow; CD36, CD36 Antigen (Collagen Type I Receptor, Thrombospondin Receptor); CHO, Chinese hamster ovary cells; CSA, Chondroitin sulfate A; FMT-HVD, Fundação de Medicina Tropical Dr. Heitor Vieira Dourado; gPv-pRBC, *P. vivax* gametocytes- parasitized red blood cells; HBMEC, Bone marrow endothelial cells; ICAM1, Intercellular Adhesion Molecule 1; NHP, non-human primate; RBC, Red blood cells; TNF, Tumor Necrosis Factor; VCAM, Vascular Cell Adhesion Molecule 1.
