# Supplementary material for: Plasmodium vivax Gametocytes Adherence to Bone Marrow Endothelial Cells
Source: Front Cell Infect Microbiol. 2021 Jun 24;11:614985. doi: 10.3389/fcimb.2021.614985 (PMC8265044; doi:10.3389/fcimb.2021.614985)
Supplement: Supplementary file 1 [file DataSheet_1.docx]

CHO cells (n=26)

**Gametocyte of *P. vivax* adhesion assay**

Isolates/Samples (n=44)

BMEC-1/CDC cells (n=18)

Excluded (n = 5)

These samples only were validated in a single condition.

With stimulation of TNF-alpha

Without stimulation of TNF-alpha

Analyzed/13 samples

Samples (n)/endothelial receptor evaluated by experiment

^K1^

^CD36^

^CD36^

^VCAM^

n=1

n=1

n=1

^745^

^K1^

^ICAM^

^CD36^

^VCAM^

^VCAM^

n=8

n=2

n=1

^ICAM^

^CD36^

^ICAM^

^CD36^

^VCAM^

^ICAM^

^745^

^VCAM^

^745^

^CD36^

^745^

n=1

n=1

n=2

n=4

n=4

**Supplementary Figure 1.** Flowchart the samples used in gametocyte of *P. vivax* adhesion assay in Human bone marrow endothelial cell (HBMEC) and Chinese Hamster Ovary Cell (CHO) K1 and transfectants whit endothelial receptor CD36, ICAM-1 and VCAM and the CSA negative CHO variant pgsA (CHO-745).

**C.**

**B.**

**A.**

**
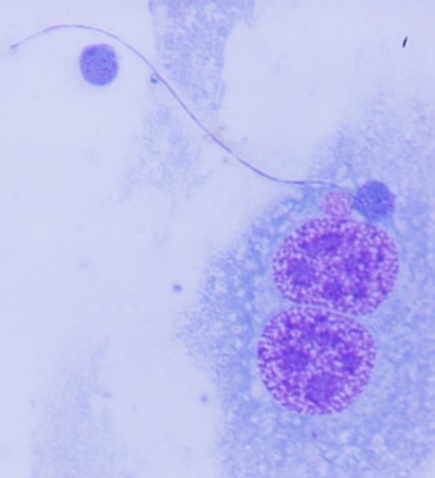

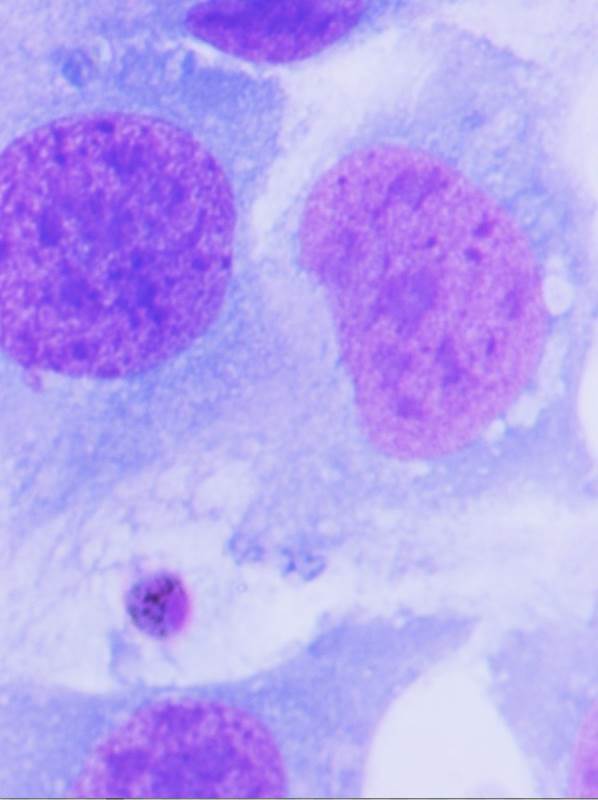

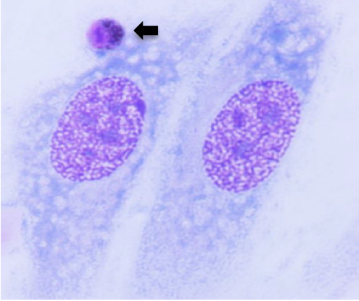
**

**
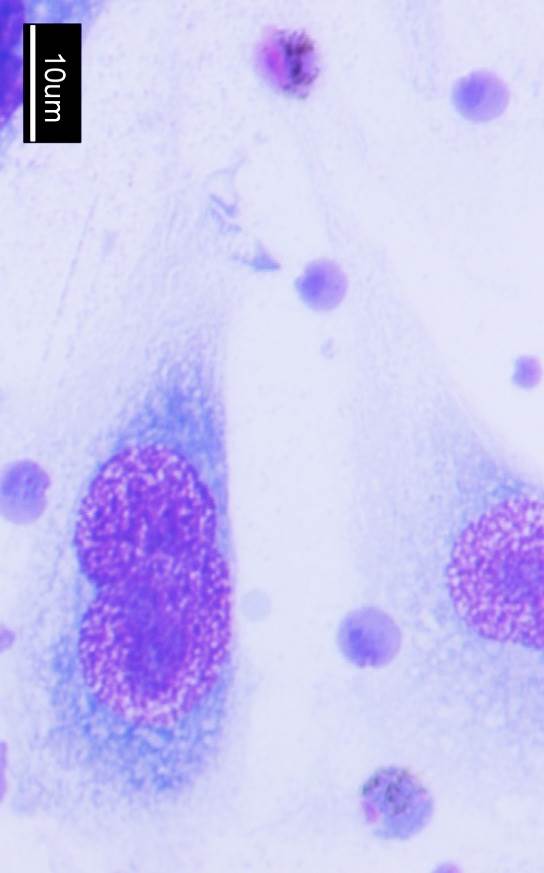
**

**E.**

**D.**


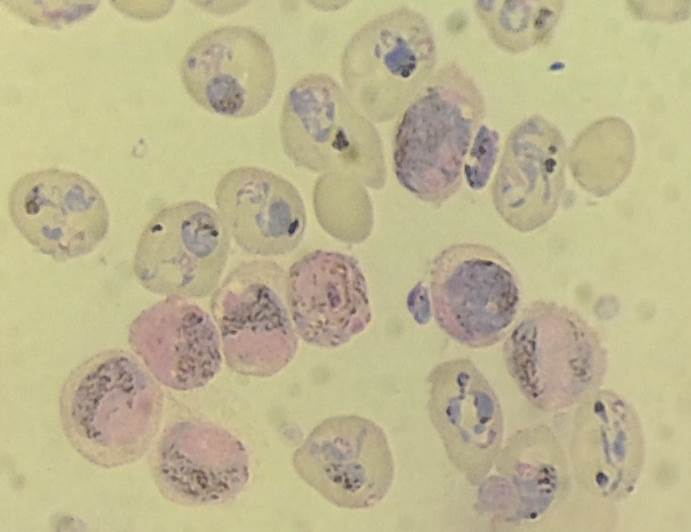


**Supplementary Figure 2. Gametocytes determination in thin smears and adhered to cell monolayer. A-C.** Photomicrographs representatives of gametocyte of *Plasmodium vivax* parasites adhered to the monolayer of bone marrow endothelial cells (HBMEC), **D.** Photomicrographs representatives of capacity to adhesion of asexual stages and gametocytes of *P. vivax* to the monolayer of HBMEC cells **E.** Parasite stage determination on thin smears after *P. vivax* gametocyte purification. Black arrows indicated gametocytes and red arrows trophozoites, the samples were stained with Giemsa and visualized using microscope at ×100 magnification.


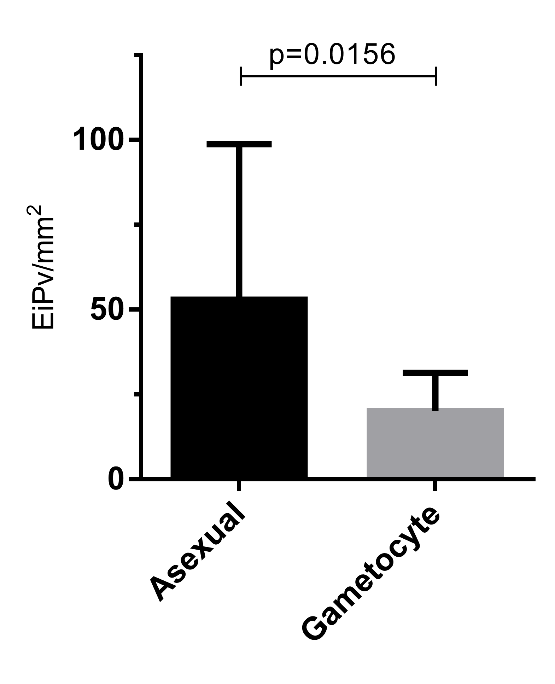


**Supplementary Figure 3.** Adhesion ability to HBMEC endothelial cells of *Plasmodium vivax* gametocytes and asexual mature stages. Data are the mean number of bound Pv-iEs per square millimeter; error bars indicate standard deviations; Wilcoxon signed-rank test (p<0.05).

**Table S1**. Number *Plasmodium vivax* gametocytes adhesion per well in HBMEC cells.

| **Samples** | **Number *Plasmodium vivax* gametocytes adhesion** | |
| --- | --- | --- |
|  | **TNF-α (-)** | **TNF-α (+)** |
| 1a | 386 | ND |
| 2a | 4 | ND |
| 3a | ND | 679 |
| 4a | 107 | ND |
| 5a | 28 | ND |
| 6a | 1 | 1 |
| 7a | 10 | 36 |
| 8a | 1 | 1 |
| 9a | 78 | 65 |
| 10a | 22 | 46 |
| 11a | 137 | 112 |
| 12a | 34 | 102 |
| 13a | 35 | 24 |
| 14a | 6 | 6 |
| 15a | 79 | 152 |
| 16a | 20 | 29 |
| 17a | 6 | 5 |
| 18a | 6 | 5 |

*ND: Not determined.

**Table S2**. Number *Plasmodium vivax* gametocytes adhesion per well in CHO-ICAM, CHO-CD36, CHO-VCAM, CHO-K1 and CHO-745 cells.

| **Samples** | **Number *Plasmodium vivax* gametocytes adhesion** | | | | |  |
| --- | --- | --- | --- | --- | --- | --- |
|  | **CHO ^745^** | **CHO ^K1^** | **CHO ^VCAM^** | **CHO ^ICAM^** | **CHO ^CD36^** | |
| 1 | 68 | 249 | ND | 226 | ND | |
| 2 | 181 | 113 | ND | 323 | ND | |
| 3 | 0 | 0 | ND | 3 | ND | |
| 4 | 0 | 17 | ND | 0 | ND | |
| 5 | 0 | 0 | ND | 0 | ND | |
| 6 | 0 | 0 | ND | 0 | ND | |
| 7 | 0 | 0 | ND | 0 | ND | |
| 8 | ND | ND | 42 | ND | 436 | |
| 9 | ND | ND | 0 | ND | ND | |
| 10 | ND | ND | 0 | ND | 12 | |
| 11 | ND | ND | ND | ND | 552 | |
| 12 | ND | ND | 37 | ND | ND | |
| 13 | ND | 249 | ND | ND | 291 | |
| 14 | 288 | 704 | ND | 380 | ND | |
| 15 | ND | ND | 438 | ND | 446 | |
| 16 | ND | ND | 413 | 930 | 383 | |
| 17 | 1 | ND | ND | 8 | ND | |
| 18 | 0 | ND | ND | 3 | ND | |
| 19 | 26 | ND | ND | ND | 184 | |
| 20 | 15 | ND | ND | ND | 21 | |
| 21 | 34 | ND | ND | ND | 49 | |
| 22 | 0 | ND | ND | ND | 32 | |
| 23 | 7 | ND | 21 | ND | ND | |
| 24 | 51 | ND | 13 | ND | ND | |
| 25 | 55 | ND | 71 | ND | ND | |
| 26 | 9 | ND | 27 | ND | ND | |

*ND: Not determined.
